# Development and validation of the Psychological Response Scale (PRS)

**DOI:** 10.1192/j.eurpsy.2025.1813

**Published:** 2025-08-26

**Authors:** S. Choi, H. Shin, S. Park

**Affiliations:** 1 Duksung Women’s University, Seoul, Korea, Republic Of

## Abstract

**Introduction:**

People experience psychological changes during adaptation process to stress; a person is angry to others or the environment but later he falls in depression. Although there are so many scales for measuring stress, they only measure the severity of stress or evaluate single emotions. None of them can track the psychological changes during the adaptation to stress.

**Objectives:**

The purpose of this study is to develop a comprehensive scale that can measure various psychological symptoms occurring in stressful situations. Specifically, the goal is to create a tool that can track an individual’s adaptation to stress over time through repeated measurements. Furthermore, the objective is to accurately understand the individual’s current state and stress adaptation patterns, enabling appropriate interventions at each stage.

**Methods:**

We established constructs and subscales based on the Kubler-Ross stages which defines general psychological change to five phase: denial, anger, bargaining, depression, acceptance. To assess the clarity and validity of the preliminary items developed, content validity was evaluated by 12 clinical psychologists and face validity was assessed with 32 adults.

**Results:**

A total of 107 initial items were developed based on literature analysis and discussions. After the content validation and face validation, items with a CVI below .83 were either revised or removed. This process resulted in a final set of 88 items: denial(18 items), anger(17 items), bargaining(19 items), depression(17 items), and acceptance(17 items). The item content validity index (I-CVI) ranged from 0.833 to 1 and the scale content validity index (S-CVI/Ave) for denial, anger, bargaining, depression and acceptance was 0.91, 0.97, 0.96, 0.98 and 0.99 respectively. In addition, exploratory and confirmatory factor analyses indicated a good level of model fit, and the internal reliability of each subscale was also found to be satisfactory.

**Conclusions:**

This results indicate that the PRS is highly valid, and that it can be utilized as an effective measure of the person’s current state. PRS can help the clinicians to understand how the patients percept the stressful situation and their stress adaptation pattern.

**Disclosure of Interest:**

None Declared

